# Modal variety of microsatellite instability in human endometrial carcinomas

**DOI:** 10.1007/s00432-015-2030-2

**Published:** 2015-08-23

**Authors:** Takako Eto, Yan Zhao, Akiko Maruyama, Kaname Miyashita, Aiko Yasui, Seiki Nakao, Kenichi Taguchi, Mototsugu Shimokawa, Shinya Oda, Toshiaki Saito

**Affiliations:** Gynecology Service, National Kyushu Cancer Center, Fukuoka, 811-1395 Japan; The Third Surgery Department, Liaoning Cancer Hospital and Institute, Shenyang, 110042 The People’s Republic of China; Department of Medicine and Bioregulatory Science, Graduate School of Medical Sciences, Kyushu University, Fukuoka, 812-8582 Japan; Clinical Research Institute, National Kyushu Cancer Center, Fukuoka, 811-1395 Japan

**Keywords:** Microsatellite instability, DNA mismatch repair, Endometrial cancer, *KRAS* mutation, Familial predisposition

## Abstract

**Purpose:**

Microsatellite instability (MSI) in human endometrial cancer (EC) was analysed using a unique fluorescent technique. MSI is associated with various human neoplasms. However, the reported frequency of MSI differs widely in each malignancy. Methodological difficulties have in fact been pointed out in its assay techniques.

**Methods:**

We previously established a sensitive fluorescent technique in which the major methodological problems are overcome. Application of this technique has revealed two distinct modes of microsatellite alterations, i.e. Type A and Type B. In the present study, we have applied this technique to 94 ECs.

**Results:**

Significant microsatellite alterations were observed in 38 (40.4 %) tumours of the panel. The two modes, Type A and Type B, were indeed observed in this malignancy. More importantly, we found that the modes more closely correlated with the molecular and clinicopathological backgrounds of the tumours than the established and widely used MSI grades, MSI-H and MSI-L. Type B MSI widely correlated with family history of hereditary non-polyposis colorectal cancer-associated cancers, whereas MSI-H only did with that of colorectal cancer. Furthermore, mutation in the *KRAS* oncogene, which has been regarded as generally infrequent in microsatellite-unstable tumours, was clearly associated with Type A MSI.

**Conclusions:**

Our observations may suggest a biological relevance and a potential utility of the modal classification of MSI and, furthermore, added complexities to genomic instability underlying tumourigenesis in human endometrium.

**Electronic supplementary material:**

The online version of this article (doi:10.1007/s00432-015-2030-2) contains supplementary material, which is available to authorized users.

## Background

Somatic instability of repetitive DNA sequences comprising minimal reiterative motifs, i.e. microsatellite instability (MSI), has initially been reported in tumours arising in Lynch syndrome [LS, *alias* hereditary non-polyposis colorectal cancer (HNPCC)] patients (Aaltonen et al. 1993), in which germline mutations in the genes functioning in DNA mismatch repair (MMR) are often found. MMR is an important cellular system that counteracts replication errors caused by DNA polymerases and, consequently, guarantees the high fidelity of DNA replication on the genome. Repetitive sequences such as microsatellites are particularly prone to replication errors, because DNA polymerases often slip on the repetitive sequences, and strand misalignment is formed. These replication errors, if uncorrected, are fixed during subsequent replication as addition or deletion of one or more repeat units. Thus, the phenomenon of MSI has been considered to reflect MMR deficiency in tumour cells. As MSI is frequently observed in various human neoplasms (Arzimanoglou et al. 1998), analyses of MSI have been prevalent in the fields of oncology or pathology. Numerous studies have been done on a wide variety of human malignancies and addressed the characteristics of MSI^+^ tumours.

**Fig. 1:**
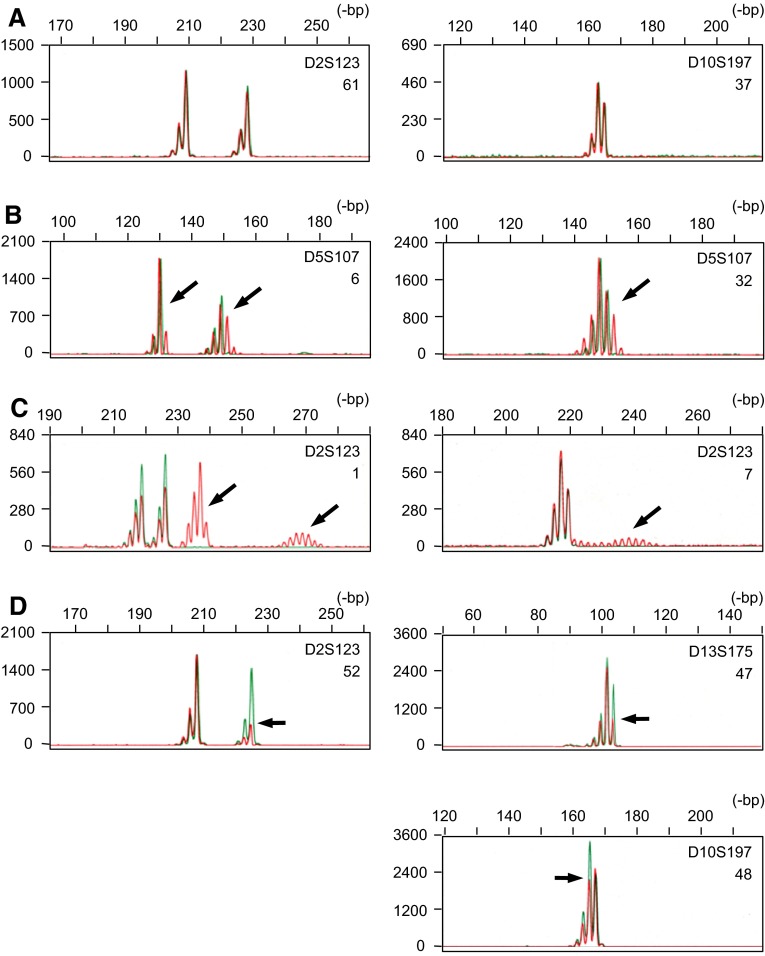
Microsatellite alterations observed in human endometrial cancer. Using genomic DNA samples prepared from tumour and the corresponding normal tissues, microsatellite sequences, indicated at the *right top* of each *panel*, were independently amplified by PCR with differentially labelled primers, then mixed and co-electrophoresed in an automated DNA sequencer. Representative results are shown and detected microsatellite alterations are indicated by *arrows*: *red lines* cancer, *green lines* normal tissues, **a** negative cases, **b** Type A MSI,** c** Type B MSI,** d** cases suspected for LOH. Data in cases of separate and inseparable heterozygous microsatellites are shown in *left* and *right* columns, respectively. Patient codes corresponding to those used in Table 1 are also indicated at the *right* of each* panel*

The reported frequency for MSI^+^ tumours in each malignancy, however, differs widely in the literature. In order to manage the confusion raised in the field, the National Cancer Institute (NCI) sponsored the workshop, ‘Microsatellite Instability and RER Phenotypes in Cancer Detection and Familial Predisposition’ (Boland et al. 1998) in 1997, which concluded that the variety of microsatellites used was a major cause of discrepancies among data from various laboratories and, consequently, recommended a panel of five microsatellites as a ‘working reference panel’. In addition, the NCI workshop recommended that the MSI^+^ phenotype should be classified into two different grades, i.e. MSI-H (high) and MSI-L (low), according to the frequencies of changes in a defined set of microsatellite markers. However, the diversity of data in the literature has not improved since then. Analysis of MSI is now commonplace, but several methodological problems have in fact been pointed out in the conventional assay techniques (Maehara et al. 2001) and may also account for the variability in results. We previously established a unique fluorescent technique designated as high-resolution fluorescent microsatellite analysis (HRFMA), in which products of polymerase chain reaction (PCR) are precisely and quantitatively resolved (Oda et al. 1997). In this technique, (a) electrophoretic profiles of microsatellites PCR products are simplified enzymatically or by primer sequence modifications, (b) each DNA fragment is detected quantitatively by use of an automated DNA sequencer, and (c) two differently labelled PCR products derived from tumour (red) and the corresponding normal tissues (green) are co-electrophoresed (see Fig. 1), in order to exclude migration errors. Application of this technique has revealed a number of previously unrecognised aspects of MSI in human cancer. In particular, we found two qualitatively distinct patterns of microsatellite alterations, i.e. Type A and Type B (Oda et al. 2005). Although this distinction has not widely been discussed, our previous data suggest that different molecular abnormalities may underlie these two modes of MSI (Oda et al. 2005). It is widely known that tumours exhibiting the MSI-H phenotype form a distinct entity with unique clinicopathological and molecular characteristics, particularly in colorectal cancer (Jass et al. 2002), and accordingly, two mutually exclusive pathways are hypothesised in colorectal tumourigenesis (Lengauer et al. 1998). Several reports, however, suggest that this distinction might be an oversimplification (Goel et al. 2003; Hawkins et al. 2001; Jass et al. 2002; Young et al. 2001). Attention has been drawn to the classification of the MSI phenotypes in human cancer.

Microsatellite alterations are most frequently observed in tumours occurring in LS kindred. More than 90 % of LS tumours are MSI^+^ (Liu et al. 1996). In the sporadic setting, endometrial carcinoma (EC) is one of the most microsatellite-unstable human neoplasms. EC indeed occurs in LS patients and is the second most frequent malignancy in LS. Numerous data have been collected on the microsatellite alterations observed in EC, and similarly to colorectal cancer, the MSI-H phenotype is now regarded as characteristic of a subset of ECs (Yeramian et al. 2013). However, also in EC, the data accumulated in the literature are diverse (see Discussion). In addition, the Type A/B classification has not been applied in assessing MSI in ECs. Using our unique dual-colour fluorescent technique (Oda et al. 1997), we analysed microsatellite alterations in detail in a relatively large panel of tumours from EC patients and found that the two modes of MSI, i.e. Type A and Type B, are indeed observed in this malignancy. More importantly, the modes more closely correlated with the clinicopathological and molecular backgrounds of the tumours than the MSI-H/L grades, which suggests that, in addition to the established classification according to the frequencies of microsatellite changes, the modal classification may also be of potential use. Here, we report the modal variety of MSI in human endometrial cancer and its biological significance.

## Methods

### Patients and tissue specimens

Samples of cancer and the corresponding normal tissues were collected from consecutive 94 EC patients who underwent surgery in Gynaecology Division of National Kyushu Cancer Center (NKCC) from 1997 to 2003. According to histopathological diagnosis, non-endometrioid-type tumours were excluded from the patient panel. This panel includes one LS patient who fulfilled Amsterdam Criteria II (Vasen et al. 1999). Specimens, taken immediately after resection, were placed in liquid nitrogen, and then stored at −80 °C.

### DNA extraction

Tissue specimens were lysed in digestion buffer (10 mM Tris-Cl pH 8.0, 0.1 M EDTA pH 8.0, 0.5 % SDS, 20 µg/ml pancreatic RNase). After treatment with proteinase K and extraction with phenol, DNA was precipitated with ethanol, then dissolved in 1X TE (10 mM Tris-Cl pH 7.5, 1 mM EDTA). The concentration of DNA was determined by OD_260_ using a spectrophotometer. The quality of DNA was checked by agarose gel electrophoresis.

### Microsatellite instability

Microsatellite analysis using fluorescence-labelled primers and an automated DNA sequencer has been described in detail (Oda et al. 1997). Briefly, five selected human dinucleotide microsatellites, D2S123, D5S107, D10S197, D11S904 and D13S175 (Oki et al. 1999), were amplified by polymerase chain reaction (PCR). Mononucleotide microsatellites were not used in order to exclude the influence of the terminal deoxynucleotidyl transferase (TDT) activity of thermostable DNA polymerases (see Discussion). Forward primers were labelled with the fluorescent compound, 6-FAM (6-carboxyfluorescein) or HEX (6-carboxy-2′, 4′, 7′, 4, 7, hexachloro-fluorescein). *TaKaRa Taq* (Takara Bio Inc., Otsu, Japan) was used as a thermostable polymerase. To compare the electrophoretic profiles between two samples, 6-FAM-labelled products and HEX-labelled products were mixed and co-electrophoresed in the ABI310 sequencer (Life Technologies, Carlsbad, CA, USA). The data were processed using the GeneScan software (Life Technologies).

### DNA sequencing

All the exons and exon-intron junctions of *MSH2* and *MLH1* were amplified by PCR using *Taq* polymerase with 3′ exonuclease activity, *TaKaRa Ex Taq*™ (Takara Bio Inc.). The sequencing strategy used is the same as the one reported by Kolodner Kolodner et al. (1994, 1995), except that the sequence complementary for M13 universal primer was deleted from each of the primer sequences, and that one-step PCR was employed. PCR products were used as a template for cycle sequencing reactions using BigDye Terminator Cycle Sequencing Kit (Life Technologies). Mutations found in one PCR product were verified by reverse sequencing and finally confirmed in two independently amplified PCR products.

The 107-bp region in the exon 2 of the *KRAS* gene that encompasses the mutation hotspots, codons 12 and 13, was amplified by PCR using ‘c-Ki-*ras*/12 primer set’ (‘c-Ki-*ras*/12 forward’, 5′-GACTGAATATAAACTTGTGG; ‘c-Ki-*ras*/12 reverse’, 5′-CTATTGTTGGATCATATTCG) (Takara Bio Inc.) and TaKaRa *Ex Taq*™ (Takara Bio Inc.). PCR products were directly used as a template for cycle sequencing reactions using BigDye terminator cycle sequencing kit (Life Technologies). The reverse primer, ‘c-Ki-*ras*/12 reverse’, was used for cycle sequencing reactions. Mutations found in one PCR product were verified by sequencing using our original inner primer that is 5′-adjacent to ‘c-Ki-*ras*/12 reverse’, KRAS2EX2R2 (5′-TCCACAAAATGATTCTGAATTAGC), and finally confirmed in three independently amplified PCR products.

### Immunohistochemistry

Tissue specimens were fixed in buffered 10 % formaldehyde and embedded in paraffin. Prior to the assay, the specimens were sectioned at 4 µm and deparaffinised using xylene. Antigen retrieval was done by heating the sections in citrate buffer. Endogenous peroxidase activity was blocked by incubation in 3.0 % H_2_O_2_. Non-specific protein binding was inhibited by incubation in 10 % normal goat serum. The sections were reacted with an adequately diluted anti-MSH2 mouse monoclonal antibody, clone GB12 (Calbiochem Novabiochem-Oncogen, Oncogen Research Products, Cambridge, MA, USA), or an anti-MLH1 antibody, clone 14 (Calbiochem Novabiochem-Oncogen, Oncogen Research Products) at 4 °C overnight. The sections were next reacted with biotinylated goat anti-mouse IgG antibody (Dako Denmark A/S, Glostrup, Denmark) and then with peroxidase-labelled streptavidin (Dako Denmark A/S). Finally, the sections were incubated with diaminobenzidine and H_2_O_2_. Counterstaining was done using Mayer’s haematoxylin. Negative control experiments were also performed by replacing the primary antibodies with a non-specific mouse IgG.

### Statistical analysis

Fisher’s exact probability test was used in all the statistical analyses.

## Results

### High-resolution fluorescent microsatellite analysis (HRFMA) reveals the modal variety of microsatellite instability in endometrial cancer

In MSI^+^ tumours, changes in microsatellite lengths are sometimes minor and as small as loss or gain of a single repeat unit, and cells carrying microsatellite changes are not always major in a given sample. However, using HRFMA, such minor and subtle alterations are sensitively and quantitatively detected, and results are highly reproducible in several independent experiments. We have applied this technique for MSI analyses in a panel of 94 ECs, in order to analyse microsatellite alterations in this malignancy in detail. The system sensitively detected various microsatellite changes. Examples are shown in Fig. 1. The basic electrophoretic profile of PCR-amplified dinucleotide microsatellite sequences is a cluster of three two base-pitched peaks. In human populations, microsatellites are highly polymorphic and, therefore, the parental alleles are different in length in many cases. In such cases, two peak clusters are separate (Fig. 1a, left) or, sometimes, partially overlap (Fig. 1a, right). Abnormal microsatellite length changes in cancer cells are detected as appearance of new peaks or changes in the peak heights in the electrophoretic profiles (Fig. 1, red lines). In the analyses of ECs, microsatellite length alterations were relatively small and within 6-bp in some cases (Fig. 1b). In the other, more drastic changes involving longer than 6-bp were also observed (Fig. 1c). We have designated the former type of microsatellite alterations as Type A and the latter as Type B in our previous observations (Oda et al. 2005). As Type B alterations involve large and, sometimes, discontinuous differences in microsatellite length, it can appear as if ‘new’ alleles, in addition to the parental alleles, are present (Fig. 1c). In EC, this type of microsatellite changes were frequently observed (Table 1), which is in clear contrast to other malignancies including colorectal cancer (Ikeda et al. 2001). In DNA fragment analyses using an automated sequencer, loss of heterozygosity (LOH) is also detectable (Fig. 1d, left), but some patterns of peak clusters are theoretically indistinguishable between MSI and LOH (Fujii et al. 2009) (Fig. 1d, right). These cases are indicated as ‘LOH’’ in Table 1. Despite minimised false positives, significant microsatellite alterations were observed in a considerable number of the subjects of the panel, and, consequently, the frequency of MSI^+^ tumours has been estimated to be 40.4% (38/94). According to the established NCI guideline, the MSI^+^ ECs were also classified into MSI-H and MSI-L (Table 1). The frequencies for MSI-H and MSI-L were 37.2 % (35/94) and 3.2 % (3/94), respectively. The relationships between MSI-H/L and Type A/B MSI are expressed in Table 2, which are highly parallel to the figure obtained in our previous observations of colorectal cancer (Ikeda et al. 2001).

**Table 1:** Microsatellite alterations observed n 94 endometrioid endometrial cancer patients

Patient code	Microsatellite					MSI A/B	MSI-H/L
	D2S	D5S	D10S	D11S	D13S		
1	B	B	B	–	B	B	H
2	B	B	B	A	–	B^a^	H
3	B	B	B	A	A	B	H
4	B	B	B	A	B	B	H
5	A	B	B	A	B	B	H
6	B	A	B	–	B	B	H
7	B	B	B	A	B	B	H
8	B	B	B	A	B	B	H
9	B	A	B	–	B	B	H
10	A	A	B	A	B	B	H
11	A	B	B	–	A	B	H
12^b^	B	B	A	A	A	B	H
13	B	A	B	–	A	B	H
14	A	B	B	–	A	B	H
15	A	B	B	A	–	B	H
16	B	A	B	A	–	B	H
17	B	–	A	–	B	B	H
18	A	B	B	A	A	B	H
19	A	A	B	A	B	B	H
20	B	A	A	–	A	B	H
21	A	A	B	A	A	B	H
22	A	A	B	A	A	B	H
23	A	A	B	A	A	B	H
24	A	B	A	A	A	B	H
25	B	A	A	A	A	B	H
26	B	A	A	A	–	B	H
27	B	A	A	–	A	B	H
28	A	A	A	–	B	B	H
29	A	A	B	–	–	B	H
30	A	B	A	–	A	B	H
31	A	A	A	–	A	A	H
32	A	A	A	A	–	A	H
33	A	A	LOH’^c^	A	A	A	H
34	A	–	–	A	A	A	H
35	A	–	A	–	–	A	H
36	A	–	–	–	–	A	L
37	A	–	–	LOH’	–	A	L
38	LOH	A	–	–	–	A	L
39		LOH’	–	–	–	N	S
40	–	–	–	–	LOH’	N	S
41	–	LOH’	LOH’	–	–	N	S
42	–	–	–	LOH’	–	N	S
43	LOH’	–	–	–	–	N	S
44	LOH’	LOH’	–	–	LOH’	N	S
45	–	LOH’	–	LOH	–	N	S
46	–	LOH	–	–	LOH’	N	S
47	–	LOH	LOH’	LOH	LOH’	N	S
48	LOH	–	LOH’	–	–	N	S
49	–	–	LOH	LOH	LOH’	N	S
50	LOH	–	LOH’	–	–	N	S
51	LOH	–	LOH’	–	–	N	S
52	LOH	–	–	LOH	LOH’	N	S
53	LOH	–	–	–	–	N	S
54	–	LOH		LOH	–	N	S
55	–	–	LOH	–	LOH	N	S
56	–	–	–	–	LOH	N	S
57	–		LOH	–	–	N	S
58	–	–	LOH	LOH	–	N	S
59	–	–	LOH	–	–	N	S
60	–	LOH		–	–	N	S
61	–	–	–	–	–	N	S
62	–	–	–	–	–	N	S
63	–	–	–	–	–	N	S
64	–	–	–	–	–	N	S
65	–	–	–	–	–	N	S
66	–	–	–	–	–	N	S
67	–	–	–	–	–	N	S
68	–	–	–	–	–	N	S
69	–	–	–	–	–	N	S
70	–	–	–	–	–	N	S
71	–	–	–	–	–	N	S
72	–	–	–	–	–	N	S
73	–	–	–	–	–	N	S
74	–	–	–	–	–	N	S
75	–	–	–	–	–	N	S
76	–	–	–	–	–	N	S
77	–	–	–	–	–	N	S
78	–	–	–	–	–	N	S
79	–	–	–	–	–	N	S
80	–	–	–	–	–	N	S
81	–	–	–	–	–	N	S
82	–	–	–	–	–	N	S
83	–	–	–	–	–	N	S
84	–	–	–	–	–	N	S
85	–	–	–	–	–	N	S
86	–	–	–	–	–	N	S
87	–	–	–	–	–	N	S
88	–	–	–	–	–	N	S
89	–	–	–	–	–	N	S
90	–	–	–	–	–	N	S
91	–	–	–	–	–	N	S
92	–	–	–	–	–	N	S
93	–	–	–	–	–	N	S
94	–	–	–	–	–	N	S

*MSI* microsatellite instability, *A* Type A MSI, *B* Type B MSI, *H* MSI-high, *L* MSI-low, *IHC* immunohistochemistry, + expressed, − no change/not expressed, *ND* not done, *LOH* loss of heterozygosity

^a^Tumours are classified as Type B when Type B alterations are observed in at least one marker

^b^The patient fulfilled Amsterdam Criteria II (Vasen et al. 1999), and a deleterious mutation (I586delT) of *MLH1* was found in the tumour. Sequence alterations were not detected in the other tumours

^c^Changes theoretically indistinguishable between MSI and LOH are indicated as ‘LOH’’ (see text)

**Table 2:** Relationship between MSI-H/L and Type A/B MSI

	Type A	Type B	Subtotal
MSI-H	5	30	35
MSI-L	3	0	3
Subtotal	8	30	38

*p* = 0.01

### Type A/B MSI characterises endometrial carcinomas with unique clinicopathological and molecular backgrounds

We next examined whether the observed microsatellite-unstable phenotypes correlated with common clinicopathological variables of the tumours. In EC, the MSI^+^ phenotype, particularly the MSI-H phenotype, has been reported to correlate with tumour grade, stage and patient survivals (see Supplementary Table). However, none of these parameters correlated with the MSI-H phenotype in this study (Table 3). Instead, we found a significant correlation with patient age and, more importantly, family history of colorectal cancer. This tendency was also confirmed in the Type A/B classification. The more important finding is that Type B MSI correlated not only with family history of colorectal cancer but also with that of ‘HNPCC-associated cancers’ (Vasen et al. 1999) (Table 4), which frequently arise in LS patients and includes, in addition to colorectal cancer and EC, carcinomas in the stomach, pancreas, small intestine, ovary and the biliary or urinary tracts and the specific types of tumours in the brain and the skin. The MSI^+^ phenotype is observed in more than 90 % of LS tumours (Liu et al. 1996) and now regarded as a molecular hallmark of LS. The strong association between Type B MSI and HNPCC-associated cancers may suggest that the Type B phenotype may better reflect the biological backgrounds of the tumours.

**Table 3:** Clinicopathological variables and the NCI classification of MSI in endometrial cancer

	MSI grade			*p* value
	MSI-L	MSI-H	MSS	
Number of cases	3	35	56	
Age				
≤55	3	23	24	0.02
≥56	0	12	32	
Stage				
1	2	24	39	0.79
2	0	0	2	
3	1	9	14	
4	0	2	1	
Grade				
1	0	14	23	0.45
2	2	12	24	
3	1	9	9	
Survival				
Alive	3	33	47	0.31
Dead	0	2	9	
Family history				
Any cancer				
Yes	1	22	30	0.54
No	2	13	26	
Colorectal cancer				
Yes	1	7	3	0.03
No	2	28	53	
Gastric cancer				
Yes	0	11	16	0.70
No	3	24	40	
HNPCC-associated cancers^a^				
Yes	1	16	16	0.20
No	2	19	40	
Double cancer				
Yes	0	4	12	0.43
No	3	31	44	
Menopause				
Before	2	12	19	0.58
After	1	23	37	

^a^Carcinomas in the colorectum, endometrium, ovary and stomach were scored as ‘HNPCC-associated cancers’ (Vasen et al. 1999). The other HNPCC-related tumours were not found in the patients’ kindred

**Table 4:** Clinicopathological variables and the MSI mode of MSI in endometrial cancer

	MSI mode			*p* value
	Type A	Type B	negative	
Number of cases	8	30	56	
Age				
<55	8	18	24	0.01
>56	0	12	32	
Stage				
1	5	21	39	0.76
2	0	0	2	
3	3	7	14	
4	0	2	1	
Grade				
1	2	12	23	0.65
2	3	11	24	
3	3	7	9	
Survival				
Alive	8	28	47	0.36
Dead	0	2	9	
Family history				
Any cancer				
Yes	3	20	30	0.26
No	5	10	26	
Colorectal cancer				
Yes	1	7	3	0.04
No	7	23	53	
Gastric cancer				
Yes	0	11	16	0.14
No	8	19	40	
HNPCC-associated cancers^a^				
Yes	1	16	16	0.03
No	7	14	40	
Double cancer				
Yes	0	4	12	0.33
No	8	26	44	
Menopause				
Before	3	11	19	0.95
After	5	19	37	

^a^Carcinomas in the colorectum, endometrium, ovary and stomach were scored as ‘HNPCC-associated cancers’ (Vasen et al. 1999). The other HNPCC-related tumours were not found in the patients’ kindred

The MSI^+^ phenotype in EC has also been reported to correlate with various gene mutations or gene expression changes in cancer cells. We therefore tested whether Type A/B phenotypes correlated with these molecular abnormalities. The *KRAS* gene is one of the most frequently mutated oncogenes in various human malignancies and is indeed known to be mutated in EC. Several early studies have reported that *KRAS* mutation was significantly associated with the MSI^+^ phenotype (Duggan et al. 1994; Lagarda et al. 2001). We therefore sequenced the genomic region of the *KRAS* gene that encompasses the most frequently mutated codons, i.e. codons 12 and 13, in the MSI^+^ tumours of our panel. *KRAS* mutation was found in five tumours. Intriguingly, *KRAS* mutation was more closely associated with Type A MSI than with MSI-H/L (Tables 5 and 6). This finding is consistent with our previous observation that *KRAS* mutation was frequently found in Type A colorectal tumours (Zhao et al. 2008). *KRAS* mutation was also frequent in tumours without family history of malignancies, although this tendency was not statistically significant (*p* = 0.07, data not shown).

**Table 5:** *KRAS* mutation and MSI-H/L

	MSI-L	MSI-H	Subtotal
KRAS			
Mutant	1	4	5
Wild type	2	31	33
Subtotal	3	35	38

*P* = 0.35

**Table 6:** KRAS mutation and Type A/B MSI

	Type A	Type B	Subtotal
KRAS			
Mutant	3	2	5
Wild type	5	28	33
Subtotal	8	30	38

*P* = 0.05

Expression of one of the essential MMR genes, *MLH1*, is known to be often lost in microsatellite-unstable ECs, primarily due to silencing by promoter methylation (Herman et al. 1998), and another essential MMR gene, *MSH2*, has also been reported to be expressed at low levels in some previous studies (Hardisson et al. 2003; Ju et al. 2006; Peiro et al. 2002). Loss of expression of these essential MMR genes causes defective MMR in cells and, consequently, leads to a destabilisation of microsatellites on the genome. Using immunohistochemistry, we examined expression of MSH2 and MLH1 proteins in the EC tissues of our panel. Although both antigens were not detected in several cases, we found that loss of MLH1 expression was observed in the majority of MSI^+^ tumours (22/36), whereas being significantly less frequent in microsatellite-stable tumours (4/51, *p* < 0.01). We further examined whether *MLH1* silencing is more closely associated with any of the MSI subcategories. However, MLH1 expression loss was similarly frequent both in Type B and in MSI-H tumours, and therefore, the correlations were not significantly different between the two classifications (data not shown). Contrary to the results of the MLH1 immunohistochemistry, MSH2 expression loss was similarly infrequent both in MSI^+^ tumours and in those with stable microsatellites and, consequently, not associated with the MSI^+^ phenotype (data not shown).

## Discussion

Numerous studies have been done on the microsatellite alterations observed in human ECs to date. Supplementary Table provides a summary of the literature. Studies of various sizes have been conducted on various subjects of different histological subtypes and genetic backgrounds, and the reported frequency for MSI^+^ tumours varies from 10 to 100 %, even if confined to the studies about endometrioid-type tumours in the sporadic setting. This variability in results may not be explicable merely from the variety of the subjects (Supplementary Table). As mentioned above, the reported frequency of MSI^+^ tumours in each malignancy is indeed diverse in the literature, and this confusion in the field has continued ever since. The variety of targets for analysis, i.e. microsatellites, will also undoubtedly lead to the variability in results. Although the NCI workshop in 1997 (Boland et al. 1998) recommended the ‘working reference panel’ comprising two mononucleotide and three dinucleotide microsatellites, various microsatellites, including tetranucleotide microsatellites, have in fact been used in the field (see Supplementary Table). Moreover, the MSI frequency differs widely even within the studies using only mononucleotide and dinucleotide microsatellites (Supplementary Table). We believe that, in addition to selection of targets for analysis, methodological problems at least partly account for the variability in results. Microsatellite length changes are sometimes as small as one or two base pairs in mononucleotide or dinucleotide microsatellites. Such small sequence alterations are not detectable in electrophoresis with significant migration errors, or under the influence of TDT activity of thermostable DNA polymerases such as *Taq*, which adds one additional base to PCR products in a sequence-dependent manner. In addition, cell populations carrying microsatellite alterations are not always predominant in a given sample. However, it is difficult to detect less abundant PCR products in an assay system using autoradiography or silver staining, due to their nonlinear detection characteristics. We previously established a sensitive fluorescent technique in which all of these methodological problems are overcome, and, in the present study, this technique was applied to address microsatellite alterations in ECs. The overall frequency of MSI^+^ tumours was 40 %, and several previous studies in the literature indeed reported similar frequencies (Furlan et al. 2006; Hardisson et al. 2003; Ohwada et al. 2002; Risinger et al. 2005) (Supplementary Table).

The MSI^+^ phenotype is now connected to specific clinicopathological characteristics of tumours. In colorectal cancer, MSI^+^ tumours more frequently occur in the proximal colon and often exhibit characteristic histopathological features such as poor/signet ring cell differentiation, mucin secretion and lymphocyte infiltration (Jass et al. 2002), which are currently known as ‘MSI-H histology’ (Umar et al. 2004). Patient outcomes are in general believed to be more favourable in MSI^+^ colorectal carcinomas (Popat et al. 2005). Accordingly, various clinicopathological features of tumours have been examined and are regarded as associated with the MSI^+^ phenotype in other human malignancies. In EC, tumour histology, grade, location, stage and patient survivals have been reported (Supplementary Table). These parameters did not correlate with MSI in this study (Tables 3, 4). The clinical and histopathological phenotypes of tumours are determined by highly complicated systems. Their relationships to MSI may therefore be more complex than hitherto suspected. MSI is now regarded as a molecular hallmark of LS. Indeed, more than 90% of LS tumours are MSI^+^ (Liu et al. 1996), which implies that MSI well reflects molecular abnormalities underlying LS tumourigenesis. One important finding of the present study is that Type B MSI did significantly correlate with family history of HNPCC-associated cancers, and that, on the other hand, MSI-H did not. This may be partly because the MSI-H phenotype sometimes includes Type A tumours (see Table 1) and inevitably tends to be heterogeneous in terms of the mode of microsatellite alterations. The tight connection between Type B MSI and LS tumourigenesis may suggest a biological importance of Type B alterations and, in addition, a potential advantage of the Type A/B classification. The MSI^+^ phenotype is also connected to various other genomic changes. Genetic instability observed in colorectal cancer has been regarded as deriving two mutually exclusive pathways, the chromosomal instability (CIN) pathway frequently associated with mutations in various oncogenes or tumour suppressors such as *TP53* and the microsatellite instability (MIN) pathway, in which *TP53* mutations are rare and, instead, mutations are found in genes harbouring mononucleotide repeats within their ORFs (Schwartz et al. 1999). The frequency for *KRAS* mutations in MSI^+^ tumours has been controversial. Mutations in the *KRAS* oncogene were initially regarded as infrequent in MSI^+^ tumours (Ionov et al. 1993; Salahshor et al. 1999; Samowitz et al. 2001). However, in our previous study, we have shown that *KRAS* mutations are indeed observed in microsatellite-unstable tumours and, more importantly, are relatively frequent in tumours exhibiting Type A MSI (Zhao et al. 2008). Also in this study, *KRAS* mutation was closely associated with Type A instability. We have previously demonstrated that Type A MSI is a direct consequence of defective MMR (Oda et al. 2005). A close association of Type A MSI with point mutations is highly consistent with the mutator phenotype in cells deficient in MMR (de Wind et al. 1995; Reitmair et al. 1997). These observations may thus suggest that the modal classification is biologically relevant.

Using HRFMA, we confirmed that endometrioid-type EC is the most microsatellite-unstable neoplasm among the human malignancies thus far tested. In general, the frequencies for MSI-H and MSI-L in colorectal cancer are 5–10 and 10–20 %, respectively. The MSI-H phenotype is very rare in the other human neoplasms, and the most frequently observed microsatellite-unstable phenotypes are generally MSI-L. Nevertheless, the MSI-H phenotype predominates in our panel of ECs (Table 1). The frequency for MSI-H exceeded 30%. The MSI-H phenotype is typical of LS (Liu et al. 1996). The high frequency of this phenotype in endometrioid-type EC may suggest that the majority of this subset of ECs arises from molecular backgrounds similar to those of LS or, in other words, via the MIN pathway. This hypothesis may be consistent with the consensus that mutations in *TP53* are relatively infrequent in endometrioid-type EC (Yeramian et al. 2013), which are similarly regarded as rare in the MIN pathway of colorectal cancer (Ionov et al. 1993; Salahshor et al. 1999; Samowitz et al. 2001; Simms et al. 1998). Tumourigenesis in this pathway has not yet been well understood. Destabilisation of repetitive motifs may disrupt the genes harbouring repeats such as *TGFBR2, IGF2R, BAX, CASP5* etc (Schwartz et al. 1999). On the other hand, mutations in established oncogenes and tumour suppressor genes and chromosomal instability leading to LOH in tumour suppressor loci are not hypothesised in the MIN pathway. Tumourigenesis without this classical model is still enigmatic. Type B MSI is observed in tumours exhibiting the MSI-H phenotype (Ikeda et al. 2001). This has been confirmed also in the present study (Table 2). Type B MSI indeed predominates in microsatellite-unstable ECs (Table 1). Our previous study suggests that defective MMR may be a promoting and, consequently, highly coincidental (as typical in Lynch syndrome patients), but insufficient factor for Type B changes, whereas being necessary and sufficient for Type A instability. In other words, whereas Type A MSI is a direct consequence of defective MMR, molecular abnormalities in addition to MMR deficiency may contribute to Type B alterations of microsatellites (Oda et al. 2005). Frequent Type B instability in endometrioid ECs suggests the possibility that this malignancy may provide a clue to Type B mechanisms and also serve as a good model for the MIN pathway tumourigenesis. Further analyses of the EC genome warrant particular attention.

## Electronic supplementary material

Supplementary material 1 (PDF 135 kb)
