# Supplementary material for: Modal variety of microsatellite instability in human endometrial carcinomas
Source: J Cancer Res Clin Oncol. 2015 Aug 23;142(2):353–63. doi: 10.1007/s00432-015-2030-2 (PMC4717170; doi:10.1007/s00432-015-2030-2)
Supplement: Supplementary file 1 — Supplementary material 1 (PDF 135 kb) [file 432_2015_2030_MOESM1_ESM.pdf]

Eto T et al.
Natl Kyushu Cancer Ctr, Japan
soda@nk-cc.go.jp

Supplementary Table Frequencies of MSI in endometrial cancer: a survey of the literature

Author	Year	Setting	Organ	Subtype	No. of patients	Marker		Method for detection	MSI[1]				Correlation with MSI
						No.	Variety		%MSI	%H[2]	%L[3]	Positive marker	
Cote ML et al.	2012	S[4]	U[5]	E[6] (55)<br>others (21)	76	10	m[7], d[8]	F[9]	18.2 (E)<br>4.8			m, d	grade, race
Zauber NP et al.	2010	S/F[10]	U	E (191)<br>others (22)	213	5	m, d	F	26.3	26.3	0		*MLH1* meth[11]
Stewart CJ et al.	2010	S	U	MELF[12] +/-	33	1	m	F-SSCP[13]	18.1	18.1	0	m	-
Koyamatsu Y et al.	2010	S	U	E	30	11	m, d	F	100	33.3	66.7		gene exp[14]
Mackay H et al.	2010	S	U	E (131)<br>others (32)	166	2	d		19.3	19.3	0	d	histol[15], DFS[16]
Murayama-Hosokawa S et al.	2010	S	U	E	42	5	m, d	F	23.8	23.8	0		-
Alves F et al.	2010	S	U	E	19	7	d	F	10.5	10.5	0	d	-
Bilbao C et al.	2010	S	U	E	93	5	m	F	22	22	0	m	stage, v inv[17],<br>DFS, DSS[18]
Kawaguchi M et al.	2009	S	U	E (59)<br>AS[19] (10)	69	5	m, d	F	31.9	31.9	0		-
Kawaguchi M et al.	2009	S	U	E (37)<br>AS (7)	44	11	m, d	F	38.6	31.8	6.8		*MLH1* meth
Ferreira AM et al.	2009	F	U	E	61	6	m, d	F	69	40	29		-
Turbiner J et al.	2008	S	U (Tam[20] +)<br>U (Tam -)	E (17)<br>C[21] (1)<br>E (22)<br>CS[22] (1)	18<br>23	2	m	F	5.6<br>34.8	5.6<br>34.8	0<br>0	m	-
Sobczuk A et al.	2007	S	U		60	5	m, d	S[23]	35	23	12		grade
Zighelboim I et al.	2007	S	U	E	446	5	m, d	R[24]	33	33	0		grade
Westin SN et al.	2008	S/F	U (lower seg)	E (27)<br>others (8)	35	6	m, d	F	34.3	34.3	0		-
Konopka B et al.	2007	S	U	E	56	9	m, d	F	46.5	28.6	17.9		-
McCourt CK et al.	2007	S	U	E (376)<br>others (69)<br>sarcoma (28)	473	5	m, d	R	32 (E)<br>10<br>21 (sarcoma)	32<br>10<br>21	0<br>0<br>0		BMI[25], race, grade,<br>histol
Ju W et al.	2006	S	U	E	50	5	m, d	F	24	24	0		MSH2/MLH1 exp
Taylor NP et al.	2006	S	U	CS	28	5	m, d	R	17.9 - 21.4	17.9 - 21.4	0		*TP53* loss, TP53 exp
Honore LH etal	2006	S	U	E	218	1	m	F	16.1	16.1	0	m	grade etc
Furlan D et al.	2006	S	synchronous O[26]+U	E	13	5	m	F	38.5	38.5	0	m	*MLH1* meth
Kang S et al.	2006	S	U		75	6	m, d		20	20	0		*MLH1* , *RASSF1A* meth
Shibata D et al.	2006	S	U		80	5	m, d	F	35	25	10		*RAB32* meth
Black D et al.	2006	S	U	E (379)<br>S[27] (71)<br>C (18)<br>others (5)	473	5	m, d	R	20	20	0		DFS, DSS
Bilbao C et al.	2006	S	U	E (182)	205	3	m	R	20	20	0	m	*PTEN* mt[28]
Chang K-H et al.	2006	S	bilat O<br>synchronous O+U	E	12<br>4	4	m, d	F	67 (O), 75 (U)	33 (O), 75 (U)	33 (O), 0 (U)	m, d	-
Irving JA et al.	2005	S	synchronous O+U	E	12	5	m, d	F	58 (O), 67 (U)	50 (O), 58 (U)	8(O), 8 (U)		-
Risinger JI et al.	2005	S	U	E	24	5	m, d	F	37.5	37.5	0		exp array profiles
Kaneki E et al.	2004	S	synchronous O+U	E	17	5	d	F	64.7 (O), 58.8 (U)	35.3(O), 35.3(U)	29.4(O), 23.5(U)	d	-
Prasad M et al.	2005	S	U	E (23)<br>MMMT[29] (3)<br>S (3)	29	7		R	24.1 - 27.6				-
Pijnenborg JMA et al.	2004	S	U	E	88	2	m	F	14.8	14.8	0	m	MLH1 exp

Eto T et al.
Natl Kyushu Cancer Ctr, Japan
soda@nk-cc.go.jp

An HJ et al.	2004	S	U	C	14	8	m, d	R	21.4	21.4	0	m, d	-
Hirasawa A et al.	2003	S	U	E	43	11	m, d	F	27.9	27.9	0		stage, grade, my inv[30]<br>N meta[31]
Hardisson D et al.	2003	S	U	E (52)<br>Non E (26)<br>HP[32] (7)	85	2	m	F	38.5 (E)<br>11.5 (Non E)<br>28.6 (HP)	38.5<br>11.5<br>28.6	0<br>0<br>0	m	histol,<br>MLH1/MSH2 exp
Miturski R et al.	2003	S	U	E (67)<br>AS (9)<br>C (3)<br>S (2)	81	1	m	S	23.9			m	-
Moreno-Bueno G et al.	2002	S	U	E (92)<br>others (33)	125	2	m	F	29.3 (E)<br>6.1	29.3<br>6.1	0<br>0	m	histol
Ferrandina G et al.	2002	S	U	E	47	6	m, d	South[33]	25.3				histol
Muresu R et al.	2002	S	U	E (67)<br>Adenoca (13)<br>S (6)	74	5	m, d	F	32				stage, grade
Baldinu P et al.	2002	S	U	E (97)<br>Adenoca (13)<br>S (6)	116	5	m, d	F	34	34	0	m, d	stage, grade
Koul A et al.	2002	S	U	E (48)<br>others (5)	53	6	m, d	R	20.8 (E)<br>20			m, d	*PTEN* mt
Peiro G et al.	2002	S	U	E (76)	89	5	m, d	F	11	11	9		stage,<br>MSH2/MLH1 exp
Machin P et al.	2002	S	U	E (59)	73	5	m, d	South	26				-
Ohwada M et al.	2002	S	U	E	66	5	d	South	40.9	40.9	0	d	-
Watanabe Y et al.	2001	S	U	E	46	7	m, d	R	17.4				T loc[34]
Moreno-Bueno G et al.	2001	S	O<br>synchronous O+U	E	26<br>4	2	m	F	19 (O)<br>100 (O), 100(U)	19<br>100 (O), 100(U)	0<br>0	m	-
Toda T et al.	2001	S	U		60	8	d	S	30	16.7	13.3	d	grade, stage
Maxwell GL et al.	2001	S	U	E	131	3	m, d, te[35]	R	22	22	0	m, d, te	TP53 exp, *PTEN* mt,<br>OS[36]
Fiumicino BS et al.	2001	S	U		65	6	m, d	South	17	17	0	m, d	grade, OS
Lagarda H et al.	2001	S	U	E (46)	58	7	m, d	South	24.1	24.1	0	m, d	*KRAS* mt
Salvesen HB et al.	2001	S	U		110	5	m, d	R	32.7	32.7	0		*PTEN* mt
Basil JB et al.	2000	S	U	E (173)<br>C (11)<br>S (10)	229	5	m, d	R	30.6	30.6	0		race, stage<br>histol
Maxwell GL et al.	2000	S	U	E (73)	100	3	m, d, te	R	15	15	0		TP53 exp, histol
MacDonald ND et al.	2000	S	U	E (234)<br>C or S (25)	259	5	m, d	R	45	45	0	m, d	-
Lax SF et al.	2000	S	U	E (57)<br>S (34)	91	8			28 (E)<br>0 (S)				histol
Ohwada M et al.	1999	S	U	E (93)	93	5	d	South	34	24	10	d	stage, N meta
Esteller M et al.	1999	S	U		27	6	m, d	S	44			m, d	*MLH1* meth
Risinger JI et al.	1998	S	U	E (115)	136	3	m, d, te		23.5	23.5	0	m, d, te	*PTEN* mt
Catasus L et al.	1998	S	U	E (33)	42	7	d	South	28	28	0	d	-
Catasus L et al.	1998	S	U	E (21)	26	5	d	South	50	50	0	d	*BAX* mt
Sakamoto T et al.	1998	S	U	E (64)<br>AS (5)<br>C (1)<br>CS (2)	72	5	d	S	14 (E)<br>40 (AS)<br>0 (C)<br>0 (CS)	14<br>40<br>0<br>0	0<br>0<br>0<br>0	d	-
Kowalski LD et al.	1997	S	U	E (80)<br>AS (12)<br>C (4)	125	7	d	R	20			d	-
Tashiro H et al.	1997	S	U	S (34)	34	8	d	R	8.8	0	8.8	d	-
Mutter GL et al.	1996	S	U	E	31	11	d	R	26			d	-
Peiffer SL etal	1995	S			37	39	d, tr[37], te	S	22	11	11	d, tr, te	-
Katabuchi H et al.	1995	S	U	S (6)<br>E (59)	65	8	d	R	18.5	18.5	0	d	-

Kobayashi K et al.	1995	S	O<br>U		145	5	d	R	9 (O)<br>54 (U)	3<br>23	6<br>31	d	grade
Duggan BD et al.	1994	S	U	E (36)<br>C (2)<br>S (7)	45	3	d	R	20	-	-	d	*KRAS* mt
Burks RT et al.	1994	S	U	E (26)<br>S (4)	30	7	d	R	23.3	23.3	0	d	-
Risinger JI et al.	1993	S<br>F	U		40	71	d	R	16.6 (S)<br>75 (F)			d	-

Abbreviations: [1]MSI = microsatellite instability; [2]H = MSI-high (H); [3]L = MSI-low (L); [4]S. sporadic; [5]U = uterus; [6]E = endometrioid; [7]m = mononucleotide; [8]d = dinucleotide; [9]F = fluorescent; [10]F = familiar; [11]meth = promoter methylation; [12]MELF = formation of microcystic = elongated and fragmented glands; [13]SSCP = single-stranded DNA conformation polymorphism; [14]exp = expression; [15]histol = histology; [16]DFS = disease-free survival; [17]v inv = vascular invasion; [18]DSS = disease-specific survival; [19]AS = adenosquamous carcinoma; [20]Tam = tamoxifen; [21]C = clear cell carcinoma; [22]CS = carcinosarcoma; [23]S = silver staining; [24]R = radioisotope; [25]BMI = body mass index; [26]O = ovary; [27]S = serous carcinoma; [28]mt = mutation; [29]MMMT = malignant mixed Mullerian tumour; [30]my inv = myometrial invasion; [31]N meta = lymphnode metastasis; [32]HP = hyperplasia; [33]South = Southern blotting; [34]T loc = tumour location; [35]te = tetranucleotide; [36]OS = overall survival; [37]tr = trinucleotide

References for Supplementary Table

Alves M, Carreira I, Liberato P, Ramos S, Mafra M, Inverno AS, Maia AT, Martins AP, Brito M, and Monteiro C (2010) Identification of a 0.4 Kb deletion region in 10q26 associated with endometrial carcinoma. Oncol Rep 23(2): 519-522

An HJ, Logani S, Isacson C, and Ellenson LH (2004) Molecular characterization of uterine clear cell carcinoma. Mod Pathol 17(5): 530-537

Baldinu P, Cossu A, Manca A, Satta MP, Pisano M, Casula M, Dessole S, Pintus A, Tanda F, and Palmieri G (2002) Microsatellite instability and mutation analysis of candidate genes in unselected sardinian patients with endometrial carcinoma. Cancer 94(12): 3157-3168

Basil JB, Goodfellow PJ, Rader JS, Mutch DG, and Herzog TJ (2000) Clinical significance of microsatellite instability in endometrial carcinoma. Cancer 89(8): 1758-1764

Bilbao C, Lara PC, Ramirez R, Henriquez-Hernandez LA, Rodriguez G, Falcon O, Leon L, Perucho M, Diaz-Chico BN, and Diaz-Chico JC (2010) Microsatellite instability predicts clinical outcome in radiation-treated endometrioid endometrial cancer. Int J Radiat Oncol Biol Phys 76(1): 9-13

Bilbao C, Rodriguez G, Ramirez R, Falcon O, Leon L, Chirino R, Rivero JF, Falcon O, Jr., Diaz-Chico BN, Diaz-Chico JC, et al (2006) The relationship between microsatellite instability and PTEN gene mutations in endometrial cancer. Int J Cancer 119(3): 563-570

Black D, Soslow RA, Levine DA, Tornos C, Chen SC, Hummer AJ, Bogomolniy F, Olvera N, Barakat RR, and Boyd J (2006) Clinicopathologic significance of defective DNA mismatch repair in endometrial carcinoma. J Clin Oncol 24(11): 1745-1753

Burks RT, Kessis TD, Cho KR, and Hedrick L (1994) Microsatellite instability in endometrial carcinoma. Oncogene 9(4): 1163-1166

Catasus L, Machin P, Matias-Guiu X, and Prat J (1998a) Microsatellite instability in endometrial carcinomas: clinicopathologic correlations in a series of 42 cases. Hum Pathol 29(10): 1160-1164

Catasus L, Matias-Guiu X, Machin P, Munoz J, and Prat J (1998b) BAX somatic frameshift mutations in endometrioid adenocarcinomas of the endometrium: evidence for a tumor progression role in endometrial carcinomas with microsatellite instability. Lab Invest 78(11): 1439-1444

Chang KH, Albarracin C, Luthra R, Wang L, Zheng W, Malpica A, Deavers MT, Silva EG, and Liu J (2006) Discordant genetic changes in ovarian and endometrial endometrioid carcinomas: a potential pitfall in molecular diagnosis. Int J Gynecol Cancer 16(1): 178-182

Cote ML, Kam A, Chang CY, Raskin L, Reding KW, Cho KR, Gruber SB, and Ali-Fehmi R (2012) A pilot study of microsatellite instability and endometrial cancer survival in white and African American women. Int J Gynecol Pathol 31(1): 66-72

Duggan BD, Felix JC, Muderspach LI, Tourgeman D, Zheng J, and Shibata D (1994) Microsatellite instability in sporadic endometrial carcinoma. J Natl Cancer Inst 86(16): 1216-1221

Esteller M, Catasus L, Matias-Guiu X, Mutter GL, Prat J, Baylin SB, and Herman JG (1999) hMLH1 promoter hypermethylation is an early event in human endometrial tumorigenesis. Am J Pathol 155(5): 1767-1772

Ferrandina G, Legge F, Ranelletti FO, Zannoni GF, Maggiano N, Evangelisti A, Mancuso S, Scambia G, and Lauriola L (2002) Cyclooxygenase-2 expression in endometrial carcinoma: correlation with clinicopathologic parameters and clinical outcome. Cancer 95(4): 801-807

Ferreira AM, Westers H, Wu Y, Niessen RC, Olderode-Berends M, van der Sluis T, van der Zee AG, Hollema H, Kleibeuker JH, Sijmons RH, et al (2009) Do microsatellite instability profiles really differ between colorectal and endometrial tumors? Genes Chromosomes Cancer 48(7): 552-557

Fiumicino S, Ercoli A, Ferrandina G, Hess P, Raspaglio G, Genuardi M, Rovella V, Bellacosa A, Cicchillitti L, Mancuso S, et al (2001) Microsatellite instability is an independent indicator of recurrence in sporadic stage I-II endometrial adenocarcinoma. J Clin Oncol 19(4): 1008-1014

Furlan D, Carnevali I, Marcomini B, Cerutti R, Dainese E, Capella C, and Riva C (2006) The high frequency of de novo promoter methylation in synchronous primary endometrial and ovarian carcinomas. Clin Cancer Res 12(11 Pt 1): 3329-3336

Hardisson D, Moreno-Bueno G, Sanchez L, Sarrio D, Suarez A, Calero F, and Palacios J (2003) Tissue microarray immunohistochemical expression analysis of mismatch repair (hMLH1 and hMSH2 genes) in endometrial carcinoma and atypical endometrial hyperplasia: relationship with microsatellite instability. Mod Pathol 16(11): 1148-1158

Hirasawa A, Aoki D, Inoue J, Imoto I, Susumu N, Sugano K, Nozawa S, and Inazawa J (2003) Unfavorable prognostic factors associated with high frequency of microsatellite instability and comparative genomic hybridization analysis in endometrial cancer. Clin Cancer Res 9(15): 5675-5682

Honore LH, Hanson J, and Andrew SE (2006) Microsatellite instability in endometrioid endometrial carcinoma: correlation with clinically relevant pathologic variables. Int J Gynecol Cancer 16(3): 1386-1392

Irving JA, Catasus L, Gallardo A, Bussaglia E, Romero M, Matias-Guiu X, and Prat J (2005) Synchronous endometrioid carcinomas of the uterine corpus and ovary: alterations in the beta-catenin (CTNNB1) pathway are associated with independent primary tumors and favorable prognosis. Hum Pathol 36(6): 605-619

Ju W, Park HM, Lee SN, Sung SH, and Kim SC (2006) Loss of hMLH1 expression is associated with less aggressive clinicopathological features in sporadic endometrioid endometrial adenocarcinoma. J Obstet Gynaecol Res 32(5): 454-460

Kaneki E, Oda Y, Ohishi Y, Tamiya S, Oda S, Hirakawa T, Nakano H, and Tsuneyoshi M (2004) Frequent microsatellite instability in synchronous ovarian and endometrial adenocarcinoma and its usefulness for differential diagnosis. Hum Pathol 35(12): 1484-1493

Kang S, Lee JM, Jeon ES, Lee S, Kim H, Kim HS, Seo SS, Park SY, Sidransky D, and Dong SM (2006) RASSF1A hypermethylation and its inverse correlation with BRAF and/or KRAS mutations in MSI-associated endometrial carcinoma. Int J Cancer 119(6): 1316-1321

Katabuchi H, van Rees B, Lambers AR, Ronnett BM, Blazes MS, Leach FS, Cho KR, and Hedrick L (1995) Mutations

in DNA mismatch repair genes are not responsible for microsatellite instability in most sporadic endometrial carcinomas. Cancer Res 55(23): 5556-5560

Kawaguchi M, Banno K, Yanokura M, Kobayashi Y, Kishimi A, Ogawa S, Kisu I, Nomura H, Hirasawa A, Susumu N, et al (2009a) Analysis of candidate target genes for mononucleotide repeat mutation in microsatellite instability-high (MSI-H) endometrial cancer. Int J Oncol 35(5): 977-982

Kawaguchi M, Yanokura M, Banno K, Kobayashi Y, Kuwabara Y, Kobayashi M, Nomura H, Hirasawa A, Susumu N, and Aoki D (2009b) Analysis of a correlation between the BRAF V600E mutation and abnormal DNA mismatch repair in patients with sporadic endometrial cancer. Int J Oncol 34(6): 1541-1547

Kobayashi K, Sagae S, Kudo R, Saito H, Koi S, and Nakamura Y (1995) Microsatellite instability in endometrial carcinomas: frequent replication errors in tumors of early onset and/or of poorly differentiated type. Genes Chromosomes Cancer 14(2): 128-132

Konopka B, Janiec-Jankowska A, Czapczak D, Paszko Z, Bidzinski M, Olszewski W, and Goluda C (2007) Molecular genetic defects in endometrial carcinomas: microsatellite instability, PTEN and beta-catenin (CTNNB1) genes mutations. J Cancer Res Clin Oncol 133(6): 361-371

Koul A, Willen R, Bendahl PO, Nilbert M, and Borg A (2002) Distinct sets of gene alterations in endometrial carcinoma implicate alternate modes of tumorigenesis. Cancer 94(9): 2369-2379

Kowalski LD, Mutch DG, Herzog TJ, Rader JS, and Goodfellow PJ (1997) Mutational analysis of MLH1 and MSH2 in 25 prospectively-acquired RER+ endometrial cancers. Genes Chromosomes Cancer 18(3): 219-227

Koyamatsu Y, Sakamoto M, Miyake K, Muroya T, Sugano K, Nakao Y, Yokoyama M, and Iwasaka T (2010) Gene expression profiles and microsatellite instability in uterine corpus endometrioid adenocarcinoma. J Obstet Gynaecol Res 36(2): 336-343

Lagarda H, Catasus L, Arguelles R, Matias-Guiu X, and Prat J (2001) K-ras mutations in endometrial carcinomas with microsatellite instability. J Pathol 193(2): 193-199

Lax SF, Kendall B, Tashiro H, Slebos RJ, and Hedrick L (2000) The frequency of p53, K-ras mutations, and microsatellite instability differs in uterine endometrioid and serous carcinoma: evidence of distinct molecular genetic pathways. Cancer 88(4): 814-824

MacDonald ND, Salvesen HB, Ryan A, Iversen OE, Akslen LA, and Jacobs IJ (2000) Frequency and prognostic impact of microsatellite instability in a large population-based study of endometrial carcinomas. Cancer Res 60(6): 1750-1752

Machin P, Catasus L, Pons C, Munoz J, Matias-Guiu X, and Prat J (2002) CTNNB1 mutations and beta-catenin expression in endometrial carcinomas. Hum Pathol 33(2): 206-212

Mackay HJ, Gallinger S, Tsao MS, McLachlin CM, Tu D, Keiser K, Eisenhauer EA, and Oza AM (2010) Prognostic value of microsatellite instability (MSI) and PTEN expression in women with endometrial cancer: results from studies of the NCIC Clinical Trials Group (NCIC CTG). Eur J Cancer 46(8): 1365-1373

Maxwell GL, Risinger JI, Alvarez AA, Barrett JC, and Berchuck A (2001) Favorable survival associated with microsatellite instability in endometrioid endometrial cancers. Obstet Gynecol 97(3): 417-422

Maxwell GL, Risinger JI, Hayes KA, Alvarez AA, Dodge RK, Barrett JC, and Berchuck A (2000) Racial disparity in the frequency of PTEN mutations, but not microsatellite instability, in advanced endometrial cancers. Clin Cancer Res 6(8): 2999-3005

McCourt CK, Mutch DG, Gibb RK, Rader JS, Goodfellow PJ, Trinkaus K, and Powell MA (2007) Body mass index: relationship to clinical, pathologic and features of microsatellite instability in endometrial cancer. Gynecol Oncol 104(3): 535-539

Miturski R, Bogusiewicz M, Tarkowski R, Ciotta C, Bignami M, Burnouf D, and Jakowicki JA (2003) BAT-26 microsatellite instability does not correlate with the loss of hMLH1 and hMSH2 protein expression in sporadic endometrial cancers. Oncol Rep 10(4): 1039-1043

Moreno-Bueno G, Gamallo C, Perez-Gallego L, de Mora JC, Suarez A, and Palacios J (2001) beta-Catenin expression pattern, beta-catenin gene mutations, and microsatellite instability in endometrioid ovarian carcinomas and synchronous endometrial carcinomas. Diagn Mol Pathol 10(2): 116-122

Moreno-Bueno G, Hardisson D, Sanchez C, Sarrio D, Cassia R, Garcia-Rostan G, Prat J, Guo M, Herman JG, Matias-Guiu X, et al (2002) Abnormalities of the APC/beta-catenin pathway in endometrial cancer. Oncogene 21(52): 7981-7990

Murayama-Hosokawa S, Oda K, Nakagawa S, Ishikawa S, Yamamoto S, Shoji K, Ikeda Y, Uehara Y, Fukayama M, McCormick F, et al (2010) Genome-wide single-nucleotide polymorphism arrays in endometrial carcinomas associate extensive chromosomal instability with poor prognosis and unveil frequent chromosomal imbalances involved in the PI3-kinase pathway. Oncogene 29(13): 1897-1908

Muresu R, Sini MC, Cossu A, Tore S, Baldinu P, Manca A, Pisano M, Loddo C, Dessole S, Pintus A, et al (2002) Chromosomal abnormalities and microsatellite instability in sporadic endometrial cancer. Eur J Cancer 38(13): 1802-1809

Mutter GL, Boynton KA, Faquin WC, Ruiz RE, and Jovanovic AS (1996) Allelotype mapping of unstable microsatellites establishes direct lineage continuity between endometrial precancers and cancer. Cancer Res 56(19): 4483-4486

Ohwada M, Suzuki M, Kashiwagi H, Konishi F, and Sato I (1999) DNA replication error in endometrial carcinoma and complex atypical endometrial hyperplasia. Cancer Genet Cytogenet 114(2): 130-135

Ohwada M, Suzuki M, Kohno T, Saga Y, Takei Y, Jobo T, Kuramoto H, and Sato I (2002) Involvement of microsatellite instability in lymph node metastasis of endometrial carcinoma. Cancer Genet Cytogenet 132(2): 152-155

Peiffer SL, Herzog TJ, Tribune DJ, Mutch DG, Gersell DJ, and Goodfellow PJ (1995) Allelic loss of sequences from the long arm of chromosome 10 and replication errors in endometrial cancers. Cancer Res 55(9): 1922-1926

Peiro G, Diebold J, Lohse P, Ruebsamen H, Baretton GB, and Lohrs U (2002) Microsatellite instability, loss of heterozygosity, and loss of hMLH1 and hMSH2 protein expression in endometrial carcinoma. Hum Pathol 33(3): 347-354

Pijnenborg JM, Dam-de Veen GC, de Haan J, van Engeland M, and Groothuis PG (2004) Defective mismatch repair and the development of recurrent endometrial carcinoma. Gynecol Oncol 94(2): 550-559

Prasad M, Wang H, Douglas W, Barakat RR, and Ellenson LH (2005) Molecular genetic characterization of tamoxifen-

associated endometrial cancer. Gynecol Oncol 96(1): 25-31

Risinger JI, Berchuck A, Kohler MF, Watson P, Lynch HT, and Boyd J (1993) Genetic instability of microsatellites in endometrial carcinoma. Cancer Res 53(21): 5100-5103

Risinger JI, Hayes K, Maxwell GL, Carney ME, Dodge RK, Barrett JC, and Berchuck A (1998) PTEN mutation in endometrial cancers is associated with favorable clinical and pathologic characteristics. Clin Cancer Res 4(12): 3005-3010

Risinger JI, Maxwell GL, Chandramouli GV, Aprelikova O, Litzi T, Umar A, Berchuck A, and Barrett JC (2005) Gene expression profiling of microsatellite unstable and microsatellite stable endometrial cancers indicates distinct pathways of aberrant signaling. Cancer Res 65(12): 5031-5037

Sakamoto T, Murase T, Urushibata H, Kato K, Takada H, Imamura T, Mori H, and Wake N (1998) Microsatellite instability and somatic mutations in endometrial carcinomas. Gynecol Oncol 71(1): 53-58

Salvesen HB, MacDonald N, Ryan A, Jacobs IJ, Lynch ED, Akslen LA, and Das S (2001) PTEN methylation is associated with advanced stage and microsatellite instability in endometrial carcinoma. Int J Cancer 91(1): 22-26

Shibata D, Mori Y, Cai K, Zhang L, Yin J, Elahi A, Hamelin R, Wong YF, Lo WK, Chung TK, et al (2006) RAB32 hypermethylation and microsatellite instability in gastric and endometrial adenocarcinomas. Int J Cancer 119(4): 801-806

Sobczuk A, Romanowicz-Makowska H, Smolarz B, and Pertynski T (2007) Microsatellite instability (MSI) and MLH1 and MSH2 protein expression analysis in postmenopausal women with sporadic endometrial cancer. J Exp Clin Cancer Res 26(3): 369-374

Stewart CJ, Amanuel B, Grieu F, Carrello A, and Iacopetta B (2010) KRAS mutation and microsatellite instability in endometrial adenocarcinomas showing MELF-type myometrial invasion. J Clin Pathol 63(7): 604-608

Tashiro H, Lax SF, Gaudin PB, Isacson C, Cho KR, and Hedrick L (1997) Microsatellite instability is uncommon in uterine serous carcinoma. Am J Pathol 150(1): 75-79

Taylor NP, Zighelboim I, Huettner PC, Powell MA, Gibb RK, Rader JS, Mutch DG, Edmonston TB, and Goodfellow PJ (2006) DNA mismatch repair and TP53 defects are early events in uterine carcinosarcoma tumorigenesis. Mod Pathol 19(10): 1333-1338

Toda T, Oku H, Khaskhely NM, Moromizato H, Ono I, and Murata T (2001) Analysis of microsatellite instability and loss of heterozygosity in uterine endometrial adenocarcinoma. Cancer Genet Cytogenet 126(2): 120-127

Turbiner J, Moreno-Bueno G, Dahiya S, Sanchez-Estevez C, Hardisson D, Prat J, Oliva E, and Palacios J (2008) Clinicopathological and molecular analysis of endometrial carcinoma associated with tamoxifen. Mod Pathol 21(8): 925-936

Watanabe Y, Nakajima H, Nozaki K, Ueda H, Obata K, Hoshiai H, and Noda K (2001) Clinicopathologic and immunohistochemical features and microsatellite status of endometrial cancer of the uterine isthmus. Int J Gynecol Pathol 20(4): 368-373

Westin SN, Lacour RA, Urbauer DL, Luthra R, Bodurka DC, Lu KH, and Broaddus RR (2008) Carcinoma of the lower uterine segment: a newly described association with Lynch syndrome. J Clin Oncol 26(36): 5965-5971

Zauber NP, Denehy TR, Taylor RR, Ongcapin EH, Marotta SP, Sabbath-Solitare M, Kulkarni R, Pradhan TS, Hermelin D, and Bishop DT (2010) Microsatellite instability and DNA methylation of endometrial tumors and clinical features in young women compared with older women. Int J Gynecol Cancer 20(9): 1549-1556

Zighelboim I, Goodfellow PJ, Gao F, Gibb RK, Powell MA, Rader JS, and Mutch DG (2007) Microsatellite instability and epigenetic inactivation of MLH1 and outcome of patients with endometrial carcinomas of the endometrioid type. J Clin Oncol 25(15): 2042-2048
